# Efficacy of Spinosad Granules and Lambda-Cyhalothrin Contrasts with Reduced Performance of Temephos for Control of *Aedes* spp. in Vehicle Tires in Veracruz, Mexico

**DOI:** 10.3390/insects10080242

**Published:** 2019-08-06

**Authors:** Trevor Williams, Juan L. Farfán, Gabriel Mercado, Javier Valle, Antonio Abella, Carlos F. Marina

**Affiliations:** 1 Instituto de Ecología AC (INECOL), Xalapa, Veracruz 91073, Mexico; 2 El Colegio de la Frontera Sur (ECOSUR), Tapachula, Chiapas 30700, Mexico; 3 Departamento de Etología, Fauna Silvestre y Animales de Laboratorio—FMVZ, Universidad Nacional Autónoma de México, Mexico City 04510, Mexico; 4 Centro Regional de Investigación en Salud Pública—INSP, Tapachula, Chiapas 30700, Mexico

**Keywords:** mosquito, vector control, larvicide, automotive tires, arbovirus, *Aedes*, *Culex*, *Toxorhynchites*, oviposition

## Abstract

The present study examined the efficacy of λ-cyhalothrin, pyriproxyfen and granular formulations of spinosad and temephos for the control of mosquito larvae present in experimental tires in Veracruz State, Mexico in the period 2015–2016. Both λ-cyhalothrin and spinosad granules provided control of larvae and pupae of *Aedes* *aegypti*, *Ae. albopictus* and *Culex* spp. in used tires in Veracruz State, Mexico, over a 9–12 week period, although numbers of *Culex* were low. The numbers of *Aedes* larvae + pupae in pyriproxyfen and temephos-treated tires were slightly less than half of the untreated control tires, probably a result the pupicidal characteristics of pyriproxyfen and possible resistance in the case of temephos. Spinosad was less harmful to predatory *Toxorhynchites* spp. than λ-cyhalothrin or temephos. The reduced susceptibility to temephos in *Aedes* populations was confirmed at five other sites in Veracruz. Public health authorities should consider incorporating spinosad as a larvicide in coastal areas at a high risk of dengue, chikungunya and Zika outbreaks in this region.

## 1. Introduction

Car and truck tires represent a highly productive habitat for the development of container-dwelling mosquitoes [1]. In developing countries, used tires are often discarded in urban areas and local tire repair businesses frequently store used tires outside where rainfall deposits and leaf detritus can contribute to the development of mosquito larvae [2,3]. Tires not only provide the shaded conditions in their interior that are favored for oviposition by many container-dwelling mosquitoes, their dark color promotes rapid warming in sunlight that hastens larval development that can advance the seasonal occurrence of vectors [4]. Tires can also remain undisturbed for long periods allowing sequential generations to reproduce continuously and reach high densities [5]. Moreover, due to the conditions they experience during larval development, mosquitoes that develop in tires may be more susceptible to infection by viruses of public health importance than individuals that develop in natural habitats [6]. In addition, the international trade in tires has been implicated in the dispersal of invasive species such as *Aedes albopictus* that likely spread to the Americas, Africa and Europe in contaminated tires [7,8]. Discarded tires, tire repair shops, and tire storage yards therefore represent localized sources of medically important mosquito species, especially in tropical urban areas.

In Mexico, the most important container dwelling mosquito vectors are *Aedes aegypti* and *Ae. albopictus*. *Aedes aegypti* is currently increasing its geographical distribution in the country under climate change [9]. This is an issue of great concern as *Ae. aegypti* is the principal vector of dengue and the arboviruses chikungunya and Zika that began to spread through Mexico in 2014 and 2015, respectively [10,11]. The Asian tiger mosquito, *Ae. albopictus* that resides in peri-domestic and peri-urban habitats, has also been firmly implicated as a vector of dengue, chikungunya and Zika [12,13]. Other mosquito species of medical importance in this region include *Culex* species, particularly *Cx. quinquefasciatus*, which can vector pathogens such as the West Nile virus, an emerging arbovirus in the Americas [14].

Vector control programs in Mexico currently focus on the elimination of oviposition sites in and around housing, in combination with the treatment of water containers with the organophosphate temephos (Abate), that is applied as a 1% mineral granule formulation. The continuous use of this compound has led to the development of resistance in many regions [15], although temephos resistance has not been reported in Mexico [16]. During outbreaks of vector borne disease, these measures are accompanied by intra-domiciliary residual spraying, street-level fogging or aerial application of insecticides, usually pyrethroids, as adulticides [17]. This dependence on a single class of insecticides has resulted in an increasing incidence of pyrethroid resistance in many mosquito species both in Mexico [18,19], and elsewhere [20].

A previous study recently tested a range of modern insecticides and identified λ-cyhalothrin and pyriproxyfen as being highly toxic to *Ae. aegypti* larvae [21]. The efficacy of these compounds as larvicides was compared with temephos granules and spinosad granules by monitoring larvicide-treated oviposition traps in an urban cemetery in southern Mexico. The pyrethroid λ-cyhalothrin targets sodium channels along the nerve axon resulting in rapid paralysis and death, whereas the organophosphate temephos is an acetylcholinesterase inhibitor [22]. Spinosad is a mixture of two naturally-derived selective neurotoxins, spinosyns A and D, that uniquely act on a subgroup of the post-synaptic nicotinic acetylcholine receptors of certain insects, including mosquitoes [23]. In contrast, as a juvenile hormone mimic, pyriproxyfen disrupts the insect endocrine system, interferes with egg hatching, larval–pupal molts, and adult eclosion and may also reduce adult fertility and longevity [24].

From the cemetery studies, it was concluded that λ-cyhalothrin and the granular formulation of spinosad could be effective for the control of *Aedes* spp. in dry and rainy seasons [21]. The findings on spinosad were consistent with our previous studies that identified liquid formulations of this naturally-derived product as effective for the control of mosquitoes in water containers [25], used tires [26] and natural habitats [27] in southern Mexico. Other studies have reported similar findings against a range of mosquito species [28,29].

The present study aimed to compare the larvicidal efficacy of λ-cyhalothrin and pyriproxyfen with that of granular formulations of spinosad and temephos in used tires in Veracruz State, Mexico. The state of Veracruz stretches 690 km along the Gulf of Mexico and is often the state with the highest number of cases of dengue in Mexico and highest number of hospitalizations due to dengue hemorrhagic fever [9]. Chikungunya and Zika are also present in this state [30,31].

## 2. Materials and Methods

### 2.1. Study Site, Tires and Insecticides

The study was performed in a radius of 50 m around an unoccupied house on the edge of the village of Tigrillos (19°22′09′′ N, 96°41′20′′ W; population ~600) in the central region of Veracruz state at an altitude of 490 m. Native trees were planted around the house and a small chicken coup with chickens was present. The climate in this region is warm and semi-humid with an average annual temperature of 25.5 °C and an annual precipitation of 900–1100 mm that mostly occurs in the period May–October.

A total of 60 discarded automotive tires for 13–15′′ diameter wheel rims were obtained from the Departamento de Regulación y Control Sanitario (H. Ayuntamiento de Xalapa, Xalapa, Mexico). These tires had been collected from around the city of Xalapa as part of a vector habitat elimination program by local public health authorities. Tires were washed inside and outside using tap water and a 5 cm diameter hole was made in the side wall of each tire using a hole cutter and electric drill.

The following insecticides were obtained from commercial suppliers: (i) A granular formulation of spinosad (Natular G30, 2.5% active ingredient (a.i.), Clarke Mosquito Control Products Inc., Roselle, IL, USA); (ii) an emulsifiable concentrate liquid formulation of pyriproxyfen (Knack CE, 11.2% a.i., Valent de México, Zapopan, Jalisco, Mexico); (iii) a suspension of λ-cyhalothrin (Karate Zeon CS, 5% a.i., Syngenta, Mexico City, Mexico); (iv) a granular mineral formulation comprising 1% a.i. temephos (Secretaría de Salud, Mexico City, Mexico). Spinosad, pyriproxyfen and temephos have been approved as larvicides in Mexico by the National Center for Preventive Programs and Disease Control [32].

### 2.2. Experimental Design and Sampling

Each of 60 tires was placed next to the trunk of a small tree (5–8 m height) surrounding the unoccupied house, over a total area of 50 × 50 m. The distance between tires was 3–6 m. Each tire was tied using a nylon rope that went around the tree trunk and the upper section of the tire to ensure that the tires remained upright during the experiment with the side wall hole located on the left hand side of the tire (at a 9 o’clock position on a clock face). Tires were placed upright to facilitate handling during sampling and to reduce the risk of spillage of water and insect larvae as tires were lifted up for weekly sampling. A 4-L volume of dechlorinated tap water was poured into each tire at the start of the experiment and a strip of filter paper (2 cm width × 15 cm length, Whatman No. 2) attached to a wooden spatula was placed resting against the inner side of each tire as an oviposition device. A number was painted in white spray paint on the upper section of each tire for identification.

The experiment was performed between 11 November, 2015 and 10 February, 2016. Pre-treatment sampling was performed on two occasions, at 8 days and 1 day prior to the application of the treatments. The sample taken at 1 day before the treatment was considered as time point zero. Sampling involved emptying the liquid in each tire through the 5 cm hole in the tire wall. The liquid was poured through a fine nylon mesh (20 cm diameter and with a pore size of ~0.4 mm) into a bucket. Mosquito larvae and pupae caught on the net were immediately placed in a white plastic tray containing water, counted, visually identified to genus (*Aedes*, *Culex* or *Toxorhynchites*) and recorded. The samples usually comprised mixtures of larval stages that were not classified by instar. A sample of 5–10 mosquito larvae from each tire was placed in a small plastic bag, labeled and taken to the laboratory in an insulated box. The remaining insects were discarded. The water from the bucket was replaced in the upright tire. The water that had evaporated during the period between samples was replaced with dechlorinated tap water to achieve a total volume of 4 L. The filter paper oviposition device was removed and replaced with a new strip. The filter paper strips were labeled and taken to the laboratory for examination.

One day after the timepoint zero pre-treatment sample had been taken, one of the following treatments was applied to 12 tires selected at random, representing 12 replicates of each treatment. The treatments were applied following federal government recommendations in Mexico [33,34]: (i) 3 granules/L of granular spinosad, equivalent to 0.2 mg a.i./L; (ii) 0.5 mg a.i./L pyriproxyfen; (iii) 1 mg a.i./L λ-cyhalothrin, tested by us in a previous study [21]; (iv) 100 mg/L of 1% temephos granules, equivalent to 1 mg a.i./L; (v) untreated water (control). To avoid loss of the granules during sampling, the spinosad and temephos granules were placed in a perforated 1.5 mL microcentrifuge tube that was removed prior to sampling and then replaced following sampling. Following the application of each treatment, tires were sampled at weekly intervals for a period of 12 weeks.

The ambient air temperature and humidity at the experimental site were measured using a digital thermometer-hygrometer (Sper Scientific, Scottsdale, AZ, USA). The temperature and humidity measurements were performed between 11:00 and 12:00 hours on each day of sampling. The weekly precipitation measurements were obtained from a weather station which was in a cement works located 2.9 km from the study site.

### 2.3. Laboratory Processing of Samples

The filter paper strips from the tire oviposition devices were examined using a dissecting microscope and the number of *Aedes* spp. eggs was counted and recorded. *Aedes* and *Culex* larvae collected from experimental tires were reared in plastic trays and held at 26 ± 1 °C, using ground laboratory rodent diet (LabDiet 500, PMI Nutrition International, St. Louis, MO, USA) as food [35]. Predatory *Toxorhynchites* larvae were not laboratory reared due to their specific nutritional requirements. Following pupation, the insects were placed in 250 mL plastic cups with 50 mL water until adult emergence. The adults were killed by freezing and then placed in a tray with silica gel until identification. The females of *Aedes* spp. were identified by patterns of the thoracic scales. For males of *Culex* spp., the genitals were removed, clarified using 10% (wt./vol.) potassium hydroxide for 24 h, mounted on an excavated microscope slide and examined. The species identification was performed with reference to published keys [36,37].

### 2.4. Studies on Larvicidal Activity of Temephos in Veracruz State

The temephos treatment of used tires did not control *Aedes* spp. larvae and pupae, suggesting that the granular temephos preparation obtained from the Secretaria de Salud had lost insecticidal activity or that the mosquito populations were no longer susceptible to this compound. To address this issue, a sample of the temephos preparation that this study had used was tested against a susceptible strain of *Ae. aegypti* (Rockefeller strain) using a colony maintained at the Centro de Investigación en Salud Pública (INSP) in Tapachula, Chiapas, Mexico, and reared following the methods described elsewhere [35]. For this, groups of 100 third instars were placed in plastic trays 35 × 25 × 5 cm that contained 1 L dechlorinated water and the quantity of temephos granules recommended for use as a larvicide (100 mg/L, equivalent to 1 mg a.i./L) [34]. The insects in the control trays were treated identically but without the presence of temephos granules. The experiment was performed with 16 trays of temephos-treated larvae and 4 trays of control larvae. After 24 h, the mortality of larvae in the treated and control trays was noted.

A brief field study was then performed in the state of Veracruz. For this, 2 L of dechlorinated water in which a small quantity of dried grass stalks had been soaked for one week was placed in each of six plastic 4-L water containers painted black on the exterior surface. The containers were placed in shaded sites in the back yard of houses of persons known to us at 11 sites: Three in the North, three in the South and five in the central region of the State, at altitudes between 5 and 1200 m above sea level (Supplementary Material, Table S1). After two weeks, the traps were examined for the presence of larvae and pupae. Three of the six containers at each site were selected at random and treated with 100 mg/L of temephos granules (1 mg a.i./L) while the other containers remained as untreated controls. Two weeks after treatment, the traps were re-examined and larvae and pupae were counted and recorded.

### 2.5. Statistical Analyses

As numbers of pupae were generally low, the counts of larvae and pupae from each tire were pooled for analysis of each genus (*Aedes*, *Culex*, *Toxorhynchites*) separately. The post-treatment numbers of larvae + pupae and egg counts from the filter papers were analyzed by fitting generalized linear mixed models with a negative binomial error distribution that accounted for the high degree of heterogeneity in the data. The significance of changes in model deviance are given in terms of χ^2^ statistics. The sample times were considered as a categorical variable. The treatments were compared by Tukey test and *z*-values. The relationships between air temperature and weekly egg counts, and between the numbers of *Toxorhynchites* spp. and other mosquito genera across all treatments, were examined using Spearman’s rank correlation. All analyses were performed using R (v. 3.4.0).

## 3. Results

The average temperature during the 12-week sampling period was 21.3 ± 0.6 °C (range 17.0–24.3 °C) and the average relative humidity was 56 ± 2% (range 51–71%). Weekly precipitation varied between 0 and 21 mm but exceeded 5 mm only in weeks 1, 7 and 12 post-treatment (Supplementary Material, Figure S1).

### 3.1. Efficacy of Larvicides

**Figure 1:**
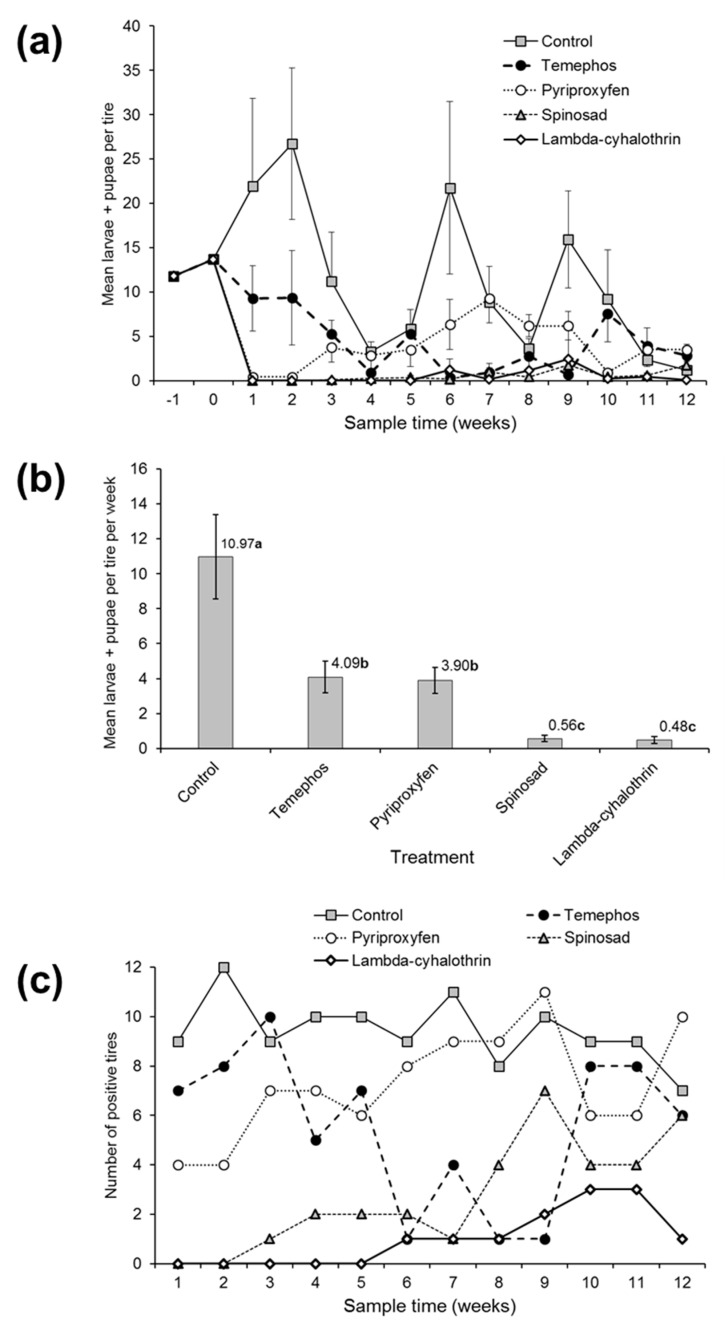
The efficacy of larvicides applied to used vehicle tires in terms of (**a**) the mean weekly counts of *Aedes* spp. larvae + pupae involving two pre-treatment samples and 12 weeks of post-treatment samples. (**b**) The overall mean numbers of *Aedes* larvae + pupae during the post-treatment period. (**c**) The number of tires in each treatment that were positive for *Aedes* larvae and pupae at each sample time (N = 12 tires per treatment). The vertical bars indicate SE. The values above columns in (**b**) indicate the mean. Values followed by identical letters did not differ significantly (Tukey, *p* > 0.05).

Pretreatment sampling indicated that the mean density of *Aedes* spp. larvae and pupae averaged 11.8–13.7 larvae + pupae per tire in the two pretreatment samples (Figure 1a). In total, 2806, 1147 and 933 larvae of *Aedes* spp., *Culex* spp. and *Toxorhynchites* spp., respectively, were counted in the experimental tires during post-treatment sampling, compared to 66, 37 and 6 pupae for each of these genera, respectively. As numbers of pupae were too low for statistical analysis, the numbers of larvae and pupae were pooled prior to analysis.

The densities of *Aedes* larvae + pupae in tires varied significantly during the 12-week post-application period (χ^2^ = 21.4, df = 11, *p* = 0.029) and differed significantly among larvicide treatments (χ^2^ = 113.5, df = 4, *p* < 0.001). The average density of *Aedes* larvae + pupae in the untreated control tires fluctuated between a weekly average of 26.8 larvae + pupae at 2 weeks post-application to 1.1 larvae + pupae in the final week of the study (Figure 1a). In contrast, following the application of treatments, the density of *Aedes* larvae + pupae was reduced to zero, or near zero, in the pyriproxyfen, spinosad granules and λ-cyhalothrin treatments, but decreased only gradually over 4 weeks in the temephos treatment (Figure 1a). The larval + pupal densities of *Aedes* spp. began to recover in the pyriproxyfen treatment from 3 weeks post-treatment whereas the mean densities in the spinosad and λ-cyhalothrin treatments remained at less than 2.5 larvae + pupae/tire for the duration of the study. Similarly, the average densities during the entire study were highest in the control (Figure 1b), lowest in the spinosad and λ-cyhalothrin treatments, and intermediate in the temephos and pyriproxyfen treatments.

In terms of the number of tires that were positive for larvae + pupae (Figure 1c), between 8 and 12 tires had *Aedes* spp. present in the control, whereas in the temephos and pyriproxyfen treatments over half the treated tires were positive for larvae or pupae at the majority of sample times. Conversely, in the spinosad treatment, less than half of the tires were positive for larvae and pupae until week 9 post-application compared with up to 3 tires (25%) in the λ-cyhalothrin treatment (Figure 1c), although as clear from the results in Figure 1a,b, only very low numbers of larvae + pupae were present in both these treatments.

Low numbers of *Culex* spp. larvae + pupae were also observed in the control and larvicide treated tires (Supplementary material, Figure S2). The numbers of *Culex* spp. larvae + pupae varied significantly among larvicide treatments (χ^2^ = 78.2, df = 4, *p* < 0.001), but did not vary significantly during the experiment (χ^2^ = 14.8, df = 11, *p* = 0.193). The mean densities were similar in the control and pyriproxyfen treatments (3.08–3.77 larvae+pupae/tire/week), compared to significantly lower values in the temephos and spinosad treatments (0.99–0.37 larvae + pupae/tire/week) that were borderline significantly different from one another (Tukey, *p* = 0.076). Larvae of *Culex* spp. were only observed on a single occasion in the λ-cyhalothrin treated tires on week 7 post-application (Supplementary Material, Figure S2). Due to the low numbers and the absence of significant changes during the sampling period, *Culex* spp. were not considered further in this study.

Laboratory rearing of larvae and pupae collected from tires (not including *Toxorhynchites* spp.) indicated that the majority of mosquitoes were *Aedes* (72%), principally *Ae. albopictus* (21%) and *Ae. aegypti* (47%) and a small number of *Aedes* (*Ochlerotatus*) *taeniorhynchus* (4%). Adults of *Culex* spp. were also present (28%), the most abundant of which were *Culex quinquefasciatus* (12%) and *Culex salinarius* (3%) (identified by examination of male genitalia). The remainder of the samples (13%) comprised unidentified specimens and a few individuals of *Wyeomyia* that were not identified to species.

### 3.2. Effect of Larvicide Treatments on Oviposition

**Figure 2:**
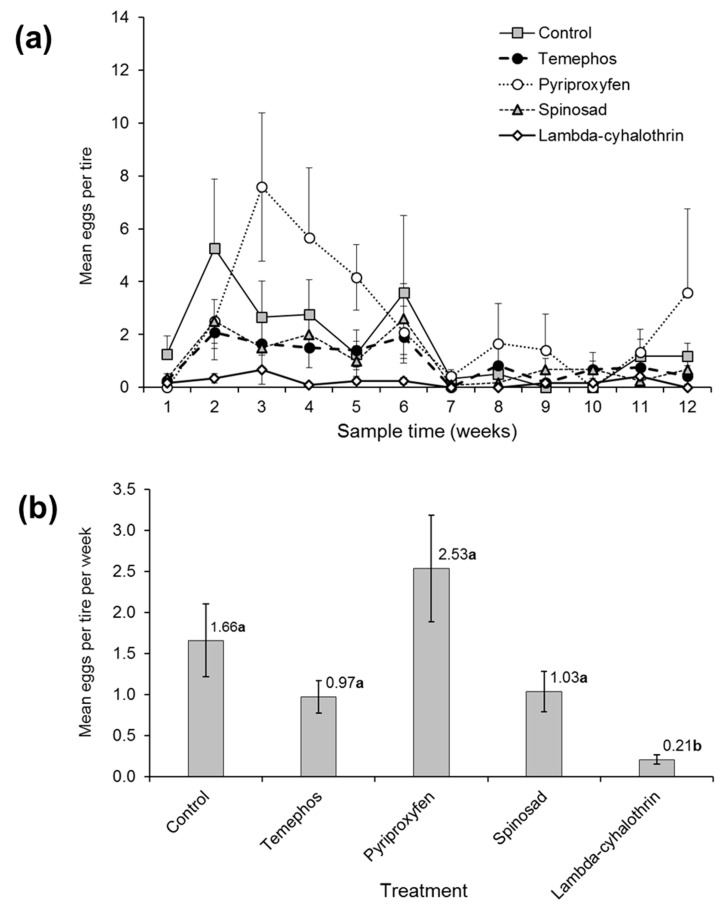
The effect of treatments on (**a**) the mean number of *Aedes* spp. eggs laid on filter paper strips in each tire during the 12-week post-treatment period. (**b**) The overall mean number of *Aedes* eggs per tire per week over the entire post-treatment period. The vertical bars indicate SE. The values above columns in (**b**) indicate the mean. Values followed by identical letters did not differ significantly (Tukey, *p* > 0.05).

The numbers of eggs laid by *Aedes* spp. on the filter paper oviposition devices placed inside each tire were quantified at weekly intervals (Figure 2a). The mean number of eggs varied significantly over time (*z* = −5.187, *p* < 0.001) and differed significantly from the control only in the λ-cyhalothrin treatment (*z* = −4.185, *p* < 0.001). A significant positive correlation was detected between the air temperature and the numbers of eggs laid across all treatments (Spearman’s rho = 0.615, *p* = 0.037). The mean numbers of eggs/tire/week varied between 0.97 and 2.53 in the temephos, pyriproxyfen and spinosad treatments which were similar to egg numbers in the control (1.66 ± 0.44) treatment, but was just 0.21 ± 0.05 eggs/tire/week in the λ-cyhalothrin treatment, suggesting that λ-cyhalothrin was deterrent for oviposition by *Aedes* spp. females (Figure 2b).

### 3.3. Influence of Larvicide Treatments on Toxorhynchites spp.

**Figure 3:**
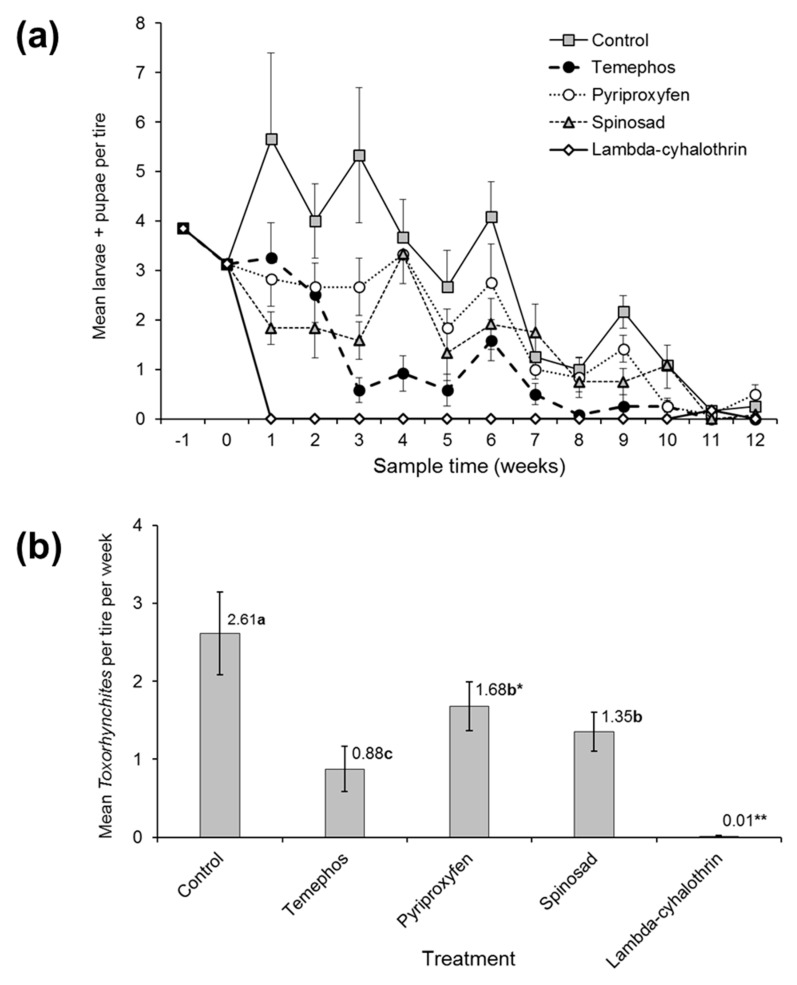
The effect of treatments on (**a**) the mean weekly counts of *Toxorhynchites* spp. larvae + pupae involving two pre-treatment samples and 12 weeks of post-treatment samples. (**b**) The overall mean numbers of *Toxorhynchites* larvae + pupae during the post-treatment period. In all cases, the vertical bars indicate SE. The values above columns in (**b**) indicate the mean. Values followed by identical letters did not differ significantly (Tukey, *p* > 0.05). * Difference between control and pyriproxyfen treatment in (**b**) was borderline significant (*p* = 0.087). ** Larvae were observed on only one occasion in the λ-cyhalothrin treatment that was excluded from the statistical analysis.

Larvae and pupae of the predatory mosquito *Toxorhynchites* spp. were present in the control and larvicide treated tires (Figure 3a). The number of *Toxorhynchites* spp. in each sample was positively correlated with the total number of *Aedes* and *Culex* larvae + pupae across all treatments (Spearman’s rho = 0.234, *p* < 0.001). The numbers of *Toxorhynchites* larvae + pupae varied significantly during the course of the experiment (χ^2^ = 210.7, df = 11, *p* < 0.001) and among larvicide treatments (χ^2^ = 39.0, df = 3, *p* < 0.001). As *Toxorhynchites* larvae were only observed on a single occasion in the λ-cyhalothrin treatment (week 11), this treatment was excluded from the analysis (Figure 3b).

The overall mean density of *Toxorhynchites* larvae + pupae was reduced by ~50% in the spinosad treatment compared to the control (*z* = −3.513, *p* = 0.002). A ~35% reduction was observed in the pyriproxyfen treatment, which was borderline significant compared to the control (*z* = −2.348, *p* = 0.087). The density of this species in the temephos treatment was significantly reduced compared to the spinosad or pyriproxyfen treatments (Figure 3b).

### 3.4. Studies on Larvicidal Activity of Temephos in Veracruz State

To determine whether the reduced efficacy of temephos observed in the experimental tires was due to a loss of larvicidal activity of the temephos preparation that was used, bioassays were performed against a colony of the Rockefeller strain of *Ae. aegypti* that is susceptible to organophosphate insecticides. The prevalence of mortality at 24 h following the temephos treatment was 99.8% (3 trays had 99 dead larvae and 13 trays had 100 dead larvae). No mortality was observed in untreated control trays. This study concluded that the 1% temephos granule preparation had not lost insecticidal activity.

**Figure 4:**
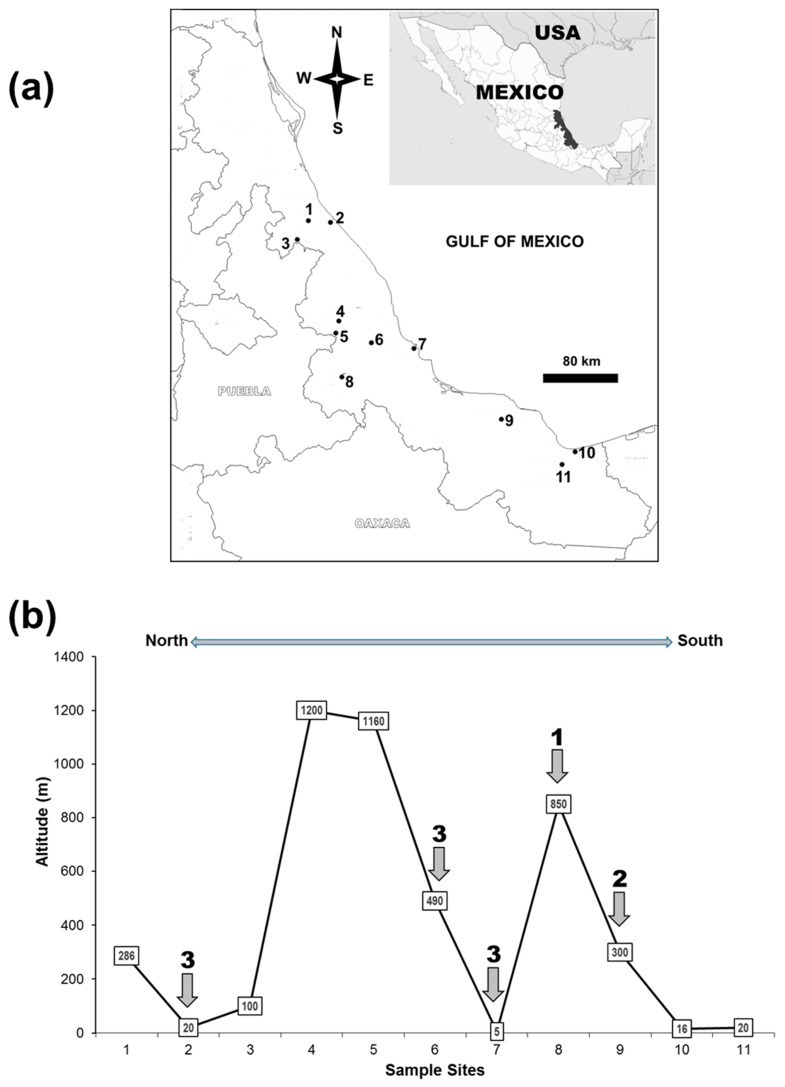
The distribution of sampling sites used to estimate susceptibility to granular temephos in the State of Veracruz, along the Gulf coast of Mexico. (**a**) Oviposition traps were placed in 11 towns and cities from North to South in the state (see Supplementary Material, Table S1). (**b**) The altitude of sites showing evidence of reduced susceptibility of *Aedes* spp. to temephos-treated oviposition traps. The sites were assessed at two weeks after oviposition traps (3 traps/site) were treated with granular temephos. The numbers above arrows indicate the number of oviposition traps that were positive for *Aedes* spp. larvae and pupae. The numbers in boxes indicate altitude in meters above mean sea level. The sites without arrows were negative for *Aedes* spp. larvae or pupae.

To determine whether the reduced efficacy of temephos was localized to Tigrillos or widely distributed, water containers were placed in the back yards of houses at 11 sites in Veracruz State (Figure 4a). After two weeks at each site, overall, 55 of 60 water containers had *Aedes* spp. larvae and pupae present (mean ± SE: 69.1 ± 6.8 larvae + pupae/container) and very low numbers of *Culex* spp. A single water container was lost at site 1 and was replaced prior to the treatment. At two weeks following temephos treatment, the water containers at five out of eleven sites (Figure 4b) had *Aedes* spp. larvae + pupae present (mean ± SE: 26.3 ± 11.6 larvae + pupae/container), despite the presence of temephos granules. These sites were distributed in along the entire length of the state at altitudes of between 5 and 890 m. All three temephos-treated containers had larvae and pupae present at sites 2, 6 and 7, whereas two containers and one container had larvae and pupae present at sites 8 and 9, respectively (indicated by arrows in Figure 4b).

## 4. Discussion

The efficacy of three modern larvicides were compared with temephos for the control of immature stages of *Aedes* spp. in used vehicle tires in the Gulf coast state of Veracruz, Mexico. The granular formulation of spinosad and the pyrethroid λ-cyhalothrin provided control of *Aedes* spp. in tires, whereas the numbers of larvae + pupae in the pyriproxyfen and temephos treatments were reduced by a little more than half compared to the control tires. Where possible, discarded vehicle tires should be removed and disposed of to reduce the risk of their use as larval mosquito habitats. However, the tires that cannot be removed or covered, such as tires in storage at roadside repair shops and in tire dumps in developing countries, represent challenging habitats to treat with larvicides because they are abundant, often remain uncovered and can serve as a source of container dwelling mosquitoes for much of the year [1].

A previously study identified pyriproxyfen and λ-cyhalothrin as highly active against *Ae. aegypti* larvae in laboratory assays of eight modern insecticides [21]. The authors went on to compare the efficacy of λ-cyhalothrin, pyriproxyfen and spinosad granules with temephos granules in an urban cemetery in the southernmost area of Mexico. Both λ-cyhalothrin and spinosad were found to be highly effective larvicides in wet and dry season trials, whereas the performance of pyriproxyfen differed in each season’s trial [21]. These findings agree well with those of the present study with 9–12 weeks of very good control of larvae and pupae by λ-cyhalothrin and spinosad, in contrast to pyriproxyfen and temephos granules that reduced immature populations of *Aedes* spp. in tires by a little over half compared to the untreated control during the study.

These findings on the sustained-release formulations of spinosad were consistent with the results of others in temperate [38] and tropical regions [39], including the Pacific coast of Mexico [40]. The larvicidal efficacy of suspension concentrate formulations of spinosad in natural and urban habitats, has been examined by the authors [25,26,27,41], and others [28,29]. The spinosad-based products in have also been tested in toxic baits and residual treatments for the control of adult mosquitoes [42,43]. Larval exposure to spinosad can have sublethal effects on the structure and function of the adult midgut and adult reproductive capacity [44,45]. Nonetheless, suitable measures should be taken to avoid the development of mosquito resistance to spinosad that has been reported in a laboratory selection study [46], and in natural populations of mosquitoes exposed to residues of spinosad used in agriculture [47].

The pyrethroid λ-cyhalothrin was selected for field testing due to its high toxicity to *Ae. aegypti* larvae in laboratory bioassays [21]. This compound was tested previously as a larvicide for the control of *Anopheles* spp. and *Culex* spp. [48,49]. The application of λ-cyhalothrin to car tires provided effective control of *Ae. albopictus* for just two weeks in Malaysia [50], compared to 24 weeks of control of *Ae. notoscriptus* in Australia [51], which may reflect the different doses used in these studies. This compound has also been successfully used for the treatment of mosquito nets [52], or for residual spraying [53,54].

In the present study, λ-cyhalothrin was the most effective compound in preventing the development of *Aedes* or *Culex* spp. in treated tires. This finding was likely due to a combination of direct toxicity to larvae and the marked deterrent effects of this compound on oviposition by *Aedes* spp. (Figure 2b). However, resistance to λ-cyhalothrin has been reported in *Ae. aegypti* populations in western Mexico [16] and Colombia [55]. To limit additional sources of selection for pyrethroid resistance in *Aedes* spp., the use of λ-cyhalothrin as a larvicide should probably be restricted to specific settings that require extended larvicidal control measures, such as car tire dumps, tire storage facilities, and similar industrial sites.

Pyriproxyfen is juvenile hormone III (JH-III) mimic and has a favorable ecotoxicological and human safety profile [56,57,58]. In the present study, the high abundance of larvae in pyriproxyfen-treated tires is likely to be related to the mode of action of this compound, which has its strongest effect as a pupicide that prevents adult emergence [59,60,61]. Indeed, in our study, numbers of pupae were low in this treatment (total of 14 *Aedes* pupae over the 12 week period). Due to this specific mode of action, numbers of larvae may be high following pyriproxyfen treatment [62]. The fact that almost no larvae or pupae were observed in the two weeks following application of pyriproxyfen may reflect the ovicidal effects of this compound that declined over the following weeks of the study (Figure 1a), as a result of its low persistence when exposed to sunlight [56]. Pyriproxyfen has ovicidal activity in the range 0.1–1 mg a.i./L, with *Ae. albopictus* being approximately twice as sensitive to this compound in the egg stage as *Ae. aegypti* [63]. Pyriproxyfen can also interrupt egg diapause in *Ae. albopictus* and appears to be more toxic to recently-laid eggs than to embrionated eggs, possibly due to the greater permeability of the egg chorion prior to embrionation [64]. Exposure to this compound results in markedly reduced female fertility or sterility [59,65]. However, monitoring of larval and pupal numbers may not have accurately reflected the efficacy of pyriproxyfen in vector control. As the presence of larvae is a strategic indicator for vector control workers, widespread adoption of this compound might compromise vector control decisions unless the monitoring of control actions is modified to include regular laboratory-rearing of larval and pupal samples from treated sites [66].

Abandoned or stored uncovered vehicle tires can be subjected to two phenomena that were not considered during the present study. First, they may undergo repeated cycles of drying and rewetting during periods of sporadic rainfall. The influence of drying and rewetting was not considered in the present study as the authors wanted to evaluate the efficacy of the products under conditions of continuous mosquito oviposition. Second, larvicides treatments applied to tires may be subjected to flushing during periods of heavy rainfall that have the potential to reduce or eliminate the active ingredients present in treated tires. Temephos and spinosad granules sink to the bottom of tires, but their efficacy over time could still be adversely affected by repeated dilution events following heavy rainfall. In the present study, rainfall was sporadic and fairly light and tires were not observed to have overflowed. Quantification of the effects of periodic flushing on larvicide persistence would require additional experiments involving known quantities of natural or artificial rainfall.

The quantification of oviposition on the filter paper strips placed in tires provided no evidence for the deterrent effects of pyriproxyfen, temephos or spinosad on *Aedes* oviposition. In line with these findings, even a very high concentration of pyriproxyfen (31 mg a.i./L) in water did not alter the oviposition responses of *Ae. aegypti* in tests performed in Peru [59]. Similarly, temephos was reported to be neither attractive nor repellent to oviposition by *Ae. albopictus* [67]. High concentrations of spinosad can be attractive to gravid *Ae. aegypti* females, but not the low concentrations used in the present study [29,68]. In contrast, a marked reduction in oviposition in λ-cyhalothrin treated tires was observed in the present study and in a previous study in southern Mexico [21], likely due to the irritant properties of pyrethroid insecticides [69].

Larval and pupal populations of predatory *Toxorhynchites* spp. were adversely affected by all the larvicides tested (Figure 3b). In the case of pyriproxyfen, larval densities were borderline significantly lower than the control, but only two pupae were observed in this treatment, possibly as a result of the developmental effects of this compound in combination with the weekly sampling frequency and the slower rate of development of *Toxorhynchites* larvae compared to *Aedes* spp. Regarding the other larvicides tested, spinosad appeared to be the least harmful, λ-cyhalothrin was the most harmful and temephos was of intermediate toxicity. It was unclear however, whether the reduced density of *Toxorhynchites* larvae in spinosad-treated tires was due to the direct toxicity of this product or due to the very low numbers of prey mosquitoes that deterred oviposition by *Toxorhynchites* females in this treatment. Overall, the prevalence of *Toxorhynchites* larvae and pupae in the control and treated tires (average 0.9–2.6 individuals/tire in all treatments, except for λ-cyhalothrin in Figure 3b) was surprising as we had not previously observed such high populations in over 15 years of testing larvicides in Mexico. However, a study on the Gulf coast of Florida reported an average density of four *T. rutilus rutilus* larvae/tire and a corresponding reduction in *Ae. aegypti* larval populations in used car tires during the fall and winter period, when the authors also performed the present study [70]. *Aedes* species differ in their sensitivity to the presence of these predators and in their susceptibility to predation when developing in the presence of *Toxorhynchites* larvae [71,72,73]. As *Toxorhynchites* spp. are considered useful biological control agents [74], the conservation of their populations through the use of selective insecticides should be considered, particularly when vector control treatments are applied to natural habitats.

Although only low numbers of *Culex* spp. were observed in the present study, the patterns of larvicide efficacy were similar to those of *Aedes* spp., with the numbers of larvae + pupae in the pyriproxyfen treatment similar to the control, whereas the abundance of larvae + pupae was markedly reduced in the spinosad and λ-cyhalothrin treatments (Supplementary Material, Figure S2). Temephos provided intermediate levels of control suggesting that species of *Culex* were more susceptible to this larvicide than *Aedes* spp., which may not be surprising as the use of temephos is mainly targeted at the control of *Aedes* spp. in this region.

The numbers of larvae + pupae in temephos-treated tires declined steadily during the first four weeks of the experiment (Figure 1a), possibly reflecting a gradual release of the active ingredient from mineral granules during this period. Similar findings were reported following the application of 1% temephos sand granules to earthenware water containers in Thailand, in which larvicidal activity increased over the 2–3 week period following application [75]. The reduced efficacy of temephos is a cause for concern as this is the principal larvicide used for the treatment of water tanks and containers that cannot be emptied or otherwise eliminated as oviposition sites. Resistance to this organophosphate has been reported in tropical regions elsewhere, including in Latin America [76] and the Caribbean [77]. Fortunately, temephos-resistant populations of *Ae. aegypti* remain highly susceptible to spinosad [78].

Only minor resistance to temephos has been reported in Mexico to date [16]. This is surprising given the extended period for which this compound has been used almost exclusively as the standard larvicide for *Aedes* control. This study detected a reduced efficacy of temephos at five localities in Veracruz State, all less than 1000 m in altitude, and including important centers of population, like the cities of Veracruz (population 437,000), Cordoba (218,000) and San Andrés (62,000), located in the central and southern parts of the state (Supplementary Material, Table S1). Veracruz is Mexico’s third most populous state, with over 8 million inhabitants, the majority of whom live in coastal areas at altitudes of less than 1000 m [79]. One of the findings of this study, therefore, is that the continued use of temephos for vector control in this region should be the subject of urgent review by public health authorities.

## 5. Conclusions

This study concludes that λ-cyhalothrin and the sustained release formulation of spinosad provided control of *Aedes* larvae and pupae in used vehicle tires at densities consistently lower than 1 individual/tire/week over a 9–12 week period. The pyriproxyfen-treated tires had high numbers of larvae, similar to those of the control tires. The immature stages of *Culex* spp. were also controlled by spinosad and λ-cyhalothrin, but numbers were generally low in both the control and larvicide treatments. Temephos and λ-cyhalothrin were more harmful to the natural mosquito predator *Toxorhynchites* than spinosad. The widely used larvicide temephos did not control *Aedes* spp., suggesting a reduced susceptibility in the *Aedes* populations, which was confirmed at various sites in the state of Veracruz. Public health authorities should consider adopting spinosad-based products, in combination with other effective larvicides, in a rotation-based system that reduces the risk of resistance to these products developing in vector populations in areas at a high risk of dengue, chikungunya and Zika outbreaks in this region.

## Supplementary Materials

The following are available online at https://www.mdpi.com/2075-4450/10/8/242/s1, Figure S1: Climatic conditions at the study site during the 12-week study; Figure S2: Mean numbers of *Culex* spp. larvae + pupae per tire per week observed over the 12-week duration of the study; Table S1: Location of oviposition traps placed at different sites in the state of Veracruz, Mexico, in June 2016.
