# Supplementary material for: Efficacy of Spinosad Granules and Lambda-Cyhalothrin Contrasts with Reduced Performance of Temephos for Control of Aedes spp. in Vehicle Tires in Veracruz, Mexico
Source: Insects. 2019 Aug 6;10(8):242. doi: 10.3390/insects10080242 (PMC6723916; doi:10.3390/insects10080242)
Supplement: Supplementary file 1 [file insects-10-00242-s001.pdf]

insects

Article

Efficacy of Spinosad Granules and Lambda-Cyhalothrin Contrasts with Reduced Performance of Temephos for Control of *Aedes* spp. in Vehicle Tires in Veracruz, Mexico

Trevor Williams [1,*], Juan L. Farfán [1], Gabriel Mercado [1], Javier Valle [2], Antonio Abella [3] and Carlos F. Marina [4]

[1] Instituto de Ecología AC (INECOL), Xalapa, 91073 Veracruz, Mexico
[2] El Colegio de la Frontera Sur (ECOSUR), Tapachula, 30700 Chiapas, Mexico
[3] Departamento de Etología, Fauna Silvestre y Animales de Laboratorio—FMVZ, Universidad Nacional Autónoma de México, Mexico City 04510, Mexico
[4] Centro Regional de Investigación en Salud Pública—INSP, Tapachula, 30700 Chiapas, Mexico
* Correspondence: trevor.williams@inecol.mx

Received: 5 June 2019; Accepted: 30 July 2019; Published: 6 August 2019

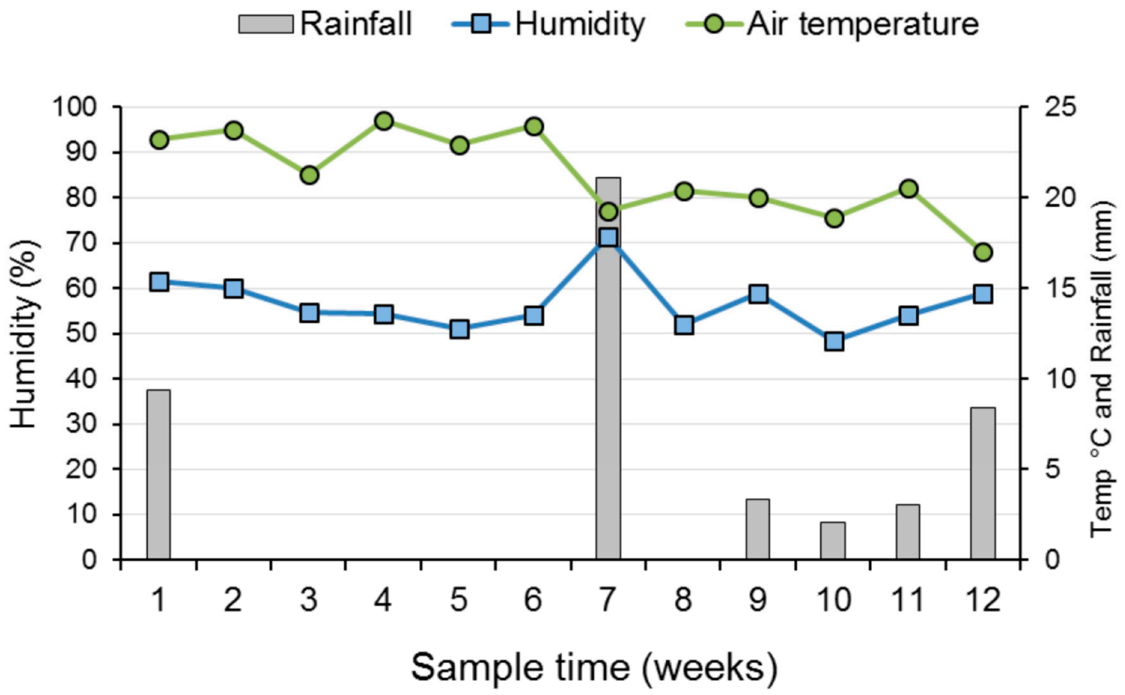

Figure S1. Climatic conditions at the study site during the12-week study. Temperature and relative humidity were measured at the moment of sampling whereas precipitation is given as a total weekly value.

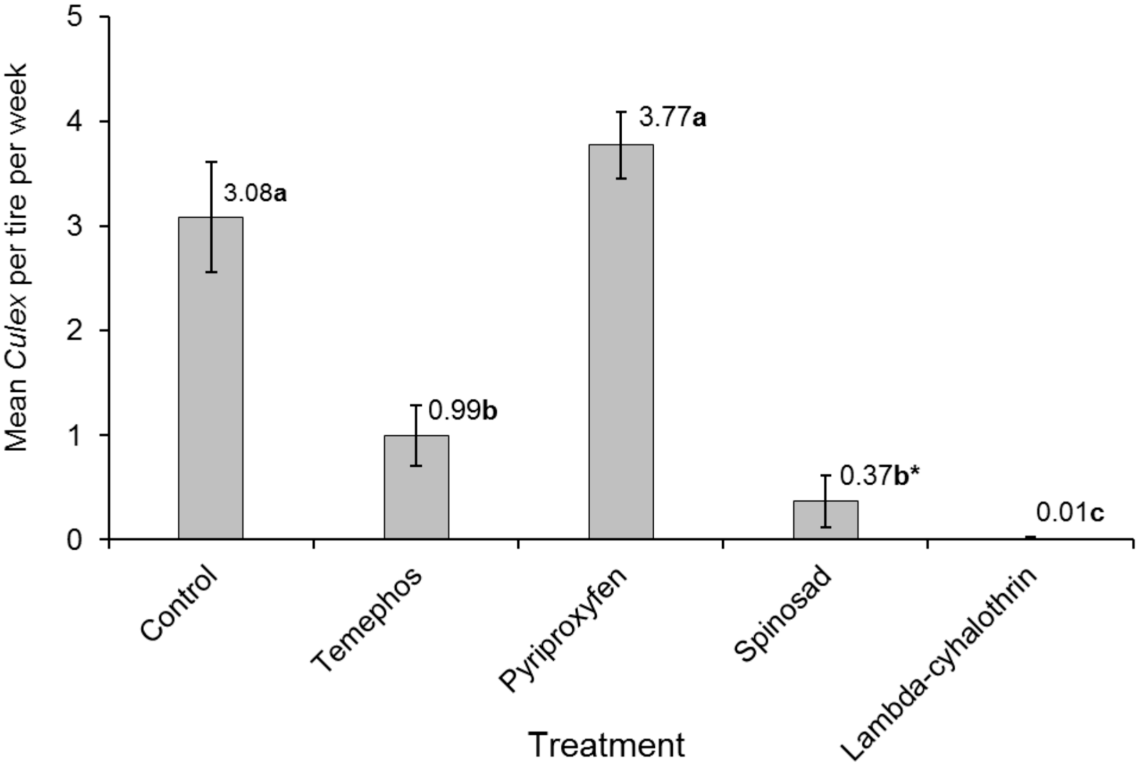

Figure S2. Mean numbers of *Culex* spp. Larvae + pupae per tire per week observed over the 12-week duration of the study. Vertical bars indicate SE. Values above columns indicate means. Values followed by identical letters did not differ significantly ($p > 0.05$). * The difference between the temephos and spinosad (NatularG30) treatments was borderline significant ($z = -2.537$, $p = 0.076$).

Table S1. Location of oviposition traps placed at different sites in the state of Veracruz, Mexico, in June 2016. Sites are arranged geographically from North to South.

Site number	Locality	Altitude (m)	Population[1]	Coordinates of oviposition traps
1	Papantla	286	161,000	20°26'52"N; 97°19'12"W
2	Gutiérrez Zamora	20	25,000	20°27'22"N; 97°05'03"W
3	Espinal	100	27,000	20°15'24"N; 97°23'45"W
4	Coatepec	1200	92,000	19°27′10"N; 96°56'00"W
5	Teocelo	1160	16,000	19°23'02"N; 96°58'08"W
6	Tigrillos	450	600	19º22'09"N, 96º41'20"W
7	Veracruz City	5	437,000	19°08'47"N; 96°07'33"W
8	Córdoba	850	218,000	18°54'19"N; 96°56'19"W
9	San Andrés Tuxtla	300	62,000	18°26'19"N; 95°11'36"W
10	Coatzacoalcos	16	319,000	18°07'58"N; 94°29'43"W
11	Minatitlán	20	356,000	18°00'02"N; 94°32'33"W

[1] 2010 population census Instituto Nacional de Estadística y Geografía (INEGI), Aguascalientes, Mexico. (http://www.beta.inegi.org.mx/contenidos/proyectos/ccpv/2010/datosabiertos/iter_30_2010_csv.zip)
